# Conducting verbal autopsy by telephone interview during the pandemic to support mortality surveillance: a feasibility study in Malaysia

**DOI:** 10.5365/wpsar.2022.13.2.902

**Published:** 2022-06-30

**Authors:** Nur Hamizah Nasaruddin, Shubash Shander Ganapathy, S. Maria Awaluddin, Mohamad Fuad Mohamad Anuar, Nazirah binti Alias, Chan Yee Mang, Khaw Wan-Fei

**Affiliations:** a Institute for Public Health, National Institutes of Health, Ministry of Health Malaysia, Selangor, Malaysia.; b Sector for Biostatistics & Data Repository, National Institutes of Health, Ministry of Health Malaysia, Selangor, Malaysia.

## Abstract

**Objective:**

Verbal autopsy (VA) through face-to-face interviews with caregivers is a way to determine cause of death without medical certification. In Malaysia, the use of VA has improved mortality statistics. However, during the coronavirus disease 2019 (COVID-19) pandemic, face-to-face interviews were delayed, reducing VA data collection and affecting data for mortality surveillance. This study aims to investigate the feasibility and acceptability of conducting VA interviews via telephone calls, and the quality of the data gathered.

**Methods:**

The study was conducted in Malaysia from September to October 2020 using a cross-sectional design. Participants were health-care workers from established VA teams across the country. They conducted VA interviews via telephone and provided feedback through a customized online form. Data collected from the form were used to assess the feasibility, acceptability and quality of the telephone interviews using IBM SPSS version 23.

**Results:**

Responses were received from 113 participants. There were 74 (65.5%) successful interviews, representing 91% of the 81 cases who were able to be contacted. More than two thirds of health-care workers provided positive feedback on the telephone interview method for themselves and the interviewees. Only 10.8% of causes of death were unusable.

**Discussion:**

This study provides preliminary evidence that VA via telephone interview is feasible, acceptable and can be used as an alternative to face-to-face interviews without affecting data quality. During times when face-to-face interviews are not advisable, VA telephone interviews can be used for data collection for mortality surveillance.

Verbal autopsy (VA) is a method developed by the World Health Organization (WHO) to determine the cause of death when medical certification is not available. (*1*) Death without medical certification usually happens at home, and the cause of death is determined by a police officer or the decedent’s caregiver. Without medical attention, the cause is often given as “old age” – such an ill-defined cause of death does not provide useful information for mortality surveillance and leads to inaccurate population health assessment. (*2*) In 2016, 47.2% of deaths registered in Malaysia were nonmedically certified deaths (NMCDs). (*3*) Reducing NMCDs would strengthen mortality statistics and contribute to better health planning. (*4*)

Malaysia incorporated VA into the death registration system in 2017 to improve mortality data. (*5*) VA is conducted via a face-to-face interview between a trained health-care worker and the decedent’s caregiver. The interviewer uses a standardized VA questionnaire to collect information on the events that led to the decedent’s death; the questionnaire is then sent to a physician for cause of death determination. (*6*-*8*) Since implementation of VA, the number of NMCDs reduced from 47.2% in 2016 to 37.2% in 2019. (*3*, *9*)

During the coronavirus disease 2019 (COVID-19) pandemic, the face-to-face VA process has been delayed due to the physical distancing preventive measures implemented. (*10*) Therefore, telephone interviews were trialled as a substitute for the standard face-to-face method because such interviews comply with the physical distancing measures of the ongoing COVID-19 pandemic. Additional benefits of a telephone interview include cost, time–effectiveness and physical anonymity, which may be appropriate given the sensitive nature of the interview questions. (*11*) These benefits, plus any challenges of using telephone interviews and whether the telephone interview method affects the quality of the data obtained from the interview, need to be investigated before implementation. Therefore, the aim of this study was to assess the feasibility, acceptability and data quality of the VA interviews when conducted via telephone in Malaysia in 2020.

## Methods

### Study design and sample selection

An exploratory cross-sectional study was conducted to determine the feasibility, acceptability and data quality of performing VA interviews via telephone. The participants for this study were health-care workers employed under Malaysia’s Ministry of Health, who were members of the District Health Office VA teams.

The sampling frame for this study was deceased individuals who died between 1 and 31 January 2020 and who were on the list of VA cases. This list was extracted from the NMCD registry, obtained from the Disease Control Division of Malaysia’s Ministry of Health. The list included the details of the deceased and the contact information of their principal caregivers. The VA cases were randomly selected to include cases from both urban and rural areas from across Malaysia. Because this was a feasibility study, 100 VA cases were selected. Each VA case was assigned to a health-care worker for a telephone-based VA interview by the coordinator of the relevant District Health Office VA team. The study team was not involved in the assignment of the VA cases to the health-care worker and had no influence on the selection. The health-care workers were identified and approached to be included in the study only after a case had been assigned to them.

### Survey process and survey instrument

For each assigned case, the health-care worker contacted the corresponding caregiver and conducted the interview with that person by telephone instead of face-to-face. The VA interview was completed according to Malaysia’s VA guidelines and procedures, using the standardized Malaysian VA questionnaire. (*6*, *7*) The health-care worker did not meet the caregiver and only interacted through the telephone call. After the interview, the health-care worker submitted the collected information to a physician in their district for cause of death determination as per the usual process. The determined cause of death was then sent to Malaysia’s Health Informatics Centre for data coding using the 10th revision of the International Classification of Diseases and Related Health Problems (ICD-10).

The health-care workers then provided their feedback on the telephone interview process, and their perception of how the caregivers reacted to being interviewed via telephone, via an online form. The form was a structured questionnaire designed in collaboration with public health experts from the Malaysian Institute for Public Health and the Disease Control Division, Ministry of Health Malaysia, and with a WHO consultant with expertise in mortality statistics, VA procedures and VA formulation in Malaysia. The questionnaire contained 53 items divided into five sections, which included the health-care worker’s characteristics, the deceased individual’s characteristics, the interview settings and outcomes, the caregiver’s characteristics and their reactions towards the telephone interview as perceived by the health-care worker, and the health-care worker’s own assessment of the telephone interview (see **Supplementary material**). This form was subsequently translated into Malay and made available online via Google Forms.

Consent from the health-care workers was obtained at the top of the online feedback form. Consent from the caregivers was only sought for the VA interview; it was obtained verbally and documented in the corresponding VA questionnaire. Further consent for the feasibility study was not warranted. Data collection was conducted between September and October 2020, resulting in a recall period of 8–9 months. Data collected from the VA questionnaire were managed according to Malaysia’s VA guidelines and procedures by the corresponding health-care workers. The data from the feedback form and the determined causes of death were compiled for analysis.

### Variable definition and analysis

#### Feasibility

The feasibility of the telephone interview was determined by the proportion of successful outcomes, defined as a complete VA questionnaire and a cause of death determined. Data from the VA telephone interview feedback form were merged with the cause of death assigned by the physicians to determine the outcome. Statistical analysis was conducted to assess the association between the interview outcomes and the characteristics of the cases and health-care workers administering the VA, and whether the call was completed using an office or personal phone.

#### Acceptability

Acceptability was assessed from the health-care workers’ feedback and their perceived reactions of the caregivers towards the telephone interview process. Among the successful outcomes, the caregivers’ perceived reactions were analysed in terms of their trust, question comprehension and cooperation throughout the telephone interview. Health-care workers’ feedback was analysed in terms of the limitations, comfort and their perceived ability to convey complicated questions during the telephone interview process.

#### Data quality

The quality of determined cause of death using ICD-10 codes was reviewed based on the proportion of causes of death without garbage code categories (a garbage code being any code that should not be the underlying cause of death, is insufficiently specified (*12*) or is unusable (*13*)). Associations between the quality of cause of death data and the health-care workers’ background were analysed by χ^2^ analysis using SPSS Statistics version 23.

## Results

A total of 116 deceased cases were selected from across Malaysia, among which VA telephone interviews were attempted for 113 (97.4%). Reasons for non-response from the remaining three cases were not documented.

### Feasibility

There were successful outcomes for 74 of 113 cases (65.5%). Of the 39 unsuccessful outcomes, seven cases (18.0%) were contactable but failed to complete the interview due to the caregiver’s distrust, disagreement or language barrier issues. Among the remaining 32 unsuccessful cases, 46.2% did not answer the call, 20.5% had incorrect telephone numbers and 15.4% did not have an available telephone number. Of the 81 cases that were contacted, 74 (91.4%) had successful outcomes.

Cases from the north-eastern zone (80.6%) had the highest number of successful outcomes, whereas the Borneo zone (45.2%) had the lowest, and the difference was significant. There was no significant difference in interview outcomes between urban and rural localities, or by the health-care workers’ sex, profession, experience with VA interviews or whether an office or personal telephone was used (**Table 1**).

**Table 1:** Characteristics of cases, health-care workers and telephone type by VA telephone interview outcomes (*n* = 113)

Characteristics	Telephone interview outcome		*P*
	Successful, *n*(%)	Unsuccessful, *n*(%)	
**Total**	74 (65.5)	39 (34.5)	-
**Cases**			
*Locality*			
Urban	40 (67.8)	19 (32.2)	0.589
Rural	34 (63.0)	20 (37.0)	-
*Zone*			
North-eastern	29 (80.6)	7 (19.4)	0.002
Central-south	26 (74.3)	9 (25.7)	-
Borneo	19 (45.2)	23 (54.8)	-
**Health-care workers**			
*Sex*			
Male	38 (63.3)	22 (36.7)	0.608
Female	36 (67.9)	17 (32.1)	-
*Profession*			
Medical officer	34 (63.0)	20 (37.0)	0.589
Medical assistant or nurse	40 (67.8)	19 (32.2)	-
*VA interview experience*			
^3^12 interviews	42 (60.0)	28 (40.0)	0.118
< 12 interviews	32 (74.4)	11 (25.6)	-
**Telephone type**			
Office telephone	41 (66.1)	21 (33.9)	0.874
Personal telephone	33 (64.7)	18 (35.3)	-

### Acceptability

The health-care workers rated most caregivers as having “easy” trust towards health-care workers, questionnaire comprehension and interview cooperation (86.5%, 87.8% and 95.9%, respectively) throughout the telephone interview (**Table 2**). A significantly higher proportion of health-care workers rated questionnaire comprehension as “difficult” for caregivers aged 60 years and over (42.9%; *P* = 0.018) (**Table 2**).

**Table 2:** Caregiver characteristics by health-care worker assessment of caregiver VA telephone interview acceptability for interviews with successful outcomes (*n* = 74)

Caregiver characteristics	Health-care worker assessment of caregiver VA telephone interview acceptability								
	Trust towards health-care worker, *n*(%)			Questionnaire comprehension, *n*(%)			Interview cooperation, *n*(%)		
	Easy	Difficult	*P*	Easy	Difficult	*P*	Good	Poor	*P*
Total	64 (86.5)	10 (13.5)	-	65 (87.8)	9 (12.2)	-	71 (95.9)	3 (4.1)	-
*Sex*									
Male	40 (81.6)	9 (18.4)	0.087	42 (85.7)	7 (14.3)	0.434	46 (93.9)	3 (6.1)	0.207
Female	24 (96.0)	1 (4.0)	-	23 (92.0)	2 (8.0)	-	25 (100.0)	0 (0.0)	-
*Age group*									
18–39 years	21 (77.8)	6 (22.2)	0.195	26 (96.3)	1 (3.7)	0.018	26 (96.3)	1 (3.7)	0.820
40–59 years	36 (90.0)	4 (10.0)	-	35 (87.5)	5 (12.5)	-	38 (95.0)	2 (5.0)	-
^3^60 years	7 (100.0)	0 (0.0)	-	4 (57.1)	3 (42.9)	-	7 (100.0)	0 (0.0)	-
*Employment status*									
White collar	17 (94.4)	1 (5.6)	0.321	18 (100.0)	0 (0.0)	0.154	18 (100.0)	0 (0.0)	0.602
Blue collar	29 (80.6)	7 (19.4)	-	31 (86.1)	5 (13.9)	-	34 (94.4)	2 (5.6)	-
Unemployed	18 (90.0)	2 (10.0)	-	16 (80.0)	4 (20.0)	-	19 (95.0)	1 (5.0)	-
*Relationship*									
Family	61 (85.9)	10 (14.1)	0.485	62 (87.3)	9 (12.7)	0.511	68 (95.8)	3 (4.2)	0.716
Non-family	3 (100.0)	0 (0.0)	-	3 (100.0)	0 (0.0)	-	3 (100.0)	0 (0.0)	-

Most health-care workers provided positive feedback towards the VA telephone interview. Most female health-care workers felt comfortable (83.3%) and found it easy to convey complicated questions (80.6%), and health-care workers from rural areas (85.3%) also felt more comfortable with telephone interviews (**Table 3**).

**Table 3:** Health-care worker characteristics by health-care worker feedback on VA telephone interview and data quality of cause of death for interviews with successful outcomes (*n* = 74)

Health-care worker characteristics	Health-care worker feedback on VA telephone interview, *n*(%)									Data quality of cause of death, *n*(%)		
	Limitations of telephone interview			Comfort of telephone interview			Ability to convey complicated questions					
	No limitation	Encountered limitation	*P*	Comfortable	Not comfortable	*P*	Easy to convey	Difficult to convey	*P*	Non-garbage code	Garbage code	*P*
Total	56 (75.7)	18 (24.3)	-	53 (71.6)	21 (28.4)	-	50 (67.6)	24 (32.4)	-	66 (89.2)	8 (10.8)	-
*Sex*												
Male	26 (68.4)	12 (31.6)	0.135	23 (60.5)	15 (39.5)	0.030	21 (55.3)	17 (44.7)	0.020	33 (86.8)	5 (13.2)	0.504
Female	30 (83.3)	6 (16.7)	-	30 (83.3)	6 (16.7)	-	29 (80.6)	7 (19.4)	-	33 (91.7)	3 (8.3)	-
*Locality*												
Urban	31 (77.5)	9 (22.5)	0.692	24 (60.0)	16 (40.0)	0.016	26 (65.0)	14 (35.0)	0.609	36 (90.0)	4 (10.0)	0.808
Rural	25 (73.5)	9 (26.5)	-	29 (85.3)	5 (14.7)	-	24 (70.6)	10 (29.4)	-	30 (88.2)	4 (11.8)	-
*Profession*												
Medical officer	27 (79.4)	7 (20.6)	0.490	27 (79.4)	7 (20.6)	0.171	26 (76.5)	8 (23.5)	0.131	30 (88.2)	4 (11.8)	0.808
Medical assistant or nurse	29 (72.5)	11 (27.5)	-	26 (65.0)	14 (35.0)	-	24 (60.0)	16 (40.0)	-	36 (90.0)	4 (10.0)	-
*VA interview experience*												
^3^12 interviews	32 (76.2)	10 (23.8)	0.906	28 (66.7)	14 (33.3)	0.279	29 (69.0)	13 (31.0)	0.755	38 (90.5)	4 (9.5)	0.683
< 12 interviews	24 (75.0)	8 (25.0)	-	25 (78.1)	7 (21.9)	-	21 (65.6)	11 (34.4)	-	28 (87.5)	4 (12.5)	-

### Data quality

There were eight cases with unusable causes of death (10.8%) that were categorized as garbage codes. The comparison between cases with and without garbage codes showed no difference between the health-care workers’ sex, locality, profession or interview experience (**Table 3**).

## Discussion

Face-to-face interview has been the standard method of communication for VA interviews. (*1*) This study shows that telephone interviews are a feasible alternative when face-to-face interviews are not possible, such as during a pandemic. (*10*) This finding aligns with multiple studies that have shown telephone interviews to be beneficial and comparable to traditional face-to-face interviews. (*11*, *14*-*16*) Telephone interviews in this study achieved a higher proportion of successful outcomes compared with a Malaysian study in 2013 of successful VA face-to-face interviews (65.5% compared with 53.1%). (*2*, *17*) That the interview outcomes were similar for both urban and rural localities suggests that telephone coverage is widely distributed across Malaysia, which may not be the case in other countries with lower urbanization levels. (*18*)

The telephone interviews for VA were acceptable in this study, with the health-care workers reporting that the interviewed caregivers showed trust, easily understood complicated questions and were cooperative throughout the interview process. Despite the presence of emotional conflicts when talking about a deceased family member, the caregivers trusted the health-care workers and were willing to complete the telephone interview. (*19*) This suggests that VA data collection is unaffected by the telephone method. The absence of obtrusive interviewer note-taking that is usually present during a face-to-face interview might have increased the focus and question comprehension of the caregiver being interviewed. (*16*)

This study did find that older caregivers encountered some difficulty in question comprehension, compared with other age groups. It is not surprising that older people had difficulties in question comprehension because this also occurs in face-to-face settings, especially for medically related questions.

Around two thirds of health-care workers provided positive feedback about conducting the VA by telephone interview. Both male and female health-care workers reported being comfortable with telephone interviews, with a higher proportion of females reporting being comfortable. This difference might be influenced by females having a lower preference for travelling and perceived interviewer safety during face-to-face interviews. Telephone interviewing reduces travelling and physical encounters with strangers outside the workplace area, which can be an issue for females. (*15*, *16*, *20*) Health-care workers from rural areas also reported being comfortable with telephone interviews, possibly due to time– and cost–effectiveness, because telephone calls make it easy to reach geographically distant caregivers in rural areas. (*11*, *16*)

Poorly collected data from a VA interview can influence a physician’s decision when determining the cause of death and lead to an ill-defined underlying cause of death or garbage code. The loss of mortality data due to unusable garbage codes is likely to affect the data quality and accuracy of mortality surveillance. (*21*) In our telephone interview study, 10.8% of cases had garbage codes, an acceptable level when compared with the 30–35% garbage codes found from a local Malaysian study involving face-to-face VA interviews. (*2*) There was no difference in data quality by the health-care workers’ specific professions and experience, suggesting that a telephone interview is easy to conduct and does not need specific skills or experience requirements.

This study highlighted a few problems with conducting VA interviews, regardless of the interview modality, such as incorrect or unavailable caregiver contact information. (*17*) A study on VA using face-to-face interviews also mentioned issues such as uncontactable caregivers due to change of address and incorrect caregiver contact information, which caused a delay in completing the interview process. (*2*, *17*) Delay between the death and the interview can make it difficult for caregivers to convey accurate information due to recall bias, especially if the delay is for more than 1 year. (*22*) Providing contact information for more than one caregiver in the civil registration system might be a potential solution for this persistent problem. Also, unanswered telephone calls, caregiver distrust and caregiver disagreement could be reduced by sending a formal letter or text message complete with organizational identification and contact information before the telephone calls to encourage people to respond to the call. (*23*)

The results from this study showed that, once a caregiver was contactable, 91% of VA interviews were successfully completed. This may be the first time the outcome of a VA telephone interview has been assessed. Participants were recruited from across all states to ensure equal distribution across the nation, and investigators were blinded from the selection of interviewers to avoid bias. Nevertheless, the study had some limitations, including a small sample size, the characteristics of unsuccessful interviews not being thoroughly investigated and the caregivers’ feedback being only from the perspective of the health-care workers.

Overall, the study found that the telephone interview method is feasible and accepted by both caregivers and health-care workers and has an acceptable level of data quality. Using this method, Malaysia could improve the VA system by incorporating the use of software for faster data collection and algorithms for automated cause of death determination. Such innovations should be explored further in future studies for Malaysia. (*24*)

## Conclusion

This study provides preliminary evidence that a VA telephone interview is feasible and can be used as an alternative to face-to-face interviews without affecting data quality or the flow of data collection. During pandemics or other instances where face-to-face interviews are not possible, the telephone interview method ensures VA data collection is not delayed and provides accuracy for mortality data in Malaysia. However, before policy decisions can be made regarding the routine use of telephone interviews, a large-scale study is recommended to yield more robust and comprehensive results to better evaluate the efficacy of telephone interviews compared with face-to-face interviews. Telephone interviews for VA should also be considered when there are transportation, geographical, time and cost limitations, and not just during the current pandemic. When feasible, these recommendations apply to other countries as well.
